# 685. Antimicrobial Stewardship Application Brings Clinical Resources to the Bedside

**DOI:** 10.1093/ofid/ofaf695.224

**Published:** 2026-01-11

**Authors:** Mandy Swann, Lauren McDaniel, Elizabeth Nowak, Nicholas Stornelli, Anthony Baffoe-Bonnie

**Affiliations:** Carilion Clinic, Roanoke, Virginia; Carilion Clinic, Roanoke, Virginia; Carilion Clinic, Roanoke, Virginia; Carilion Clinic, Roanoke, Virginia; Central Virginia VA Healthcare System

## Abstract

**Background:**

Clinicians need greater access to diagnostic and educational resources to effectively practice antimicrobial stewardship (AMS). A large medical system in Virginia had many AMS resources available but they were not accessed routinely. We describe the development of an AMS mobile application (app) to improve utilization of clinical AMS resources.

**Figure:**
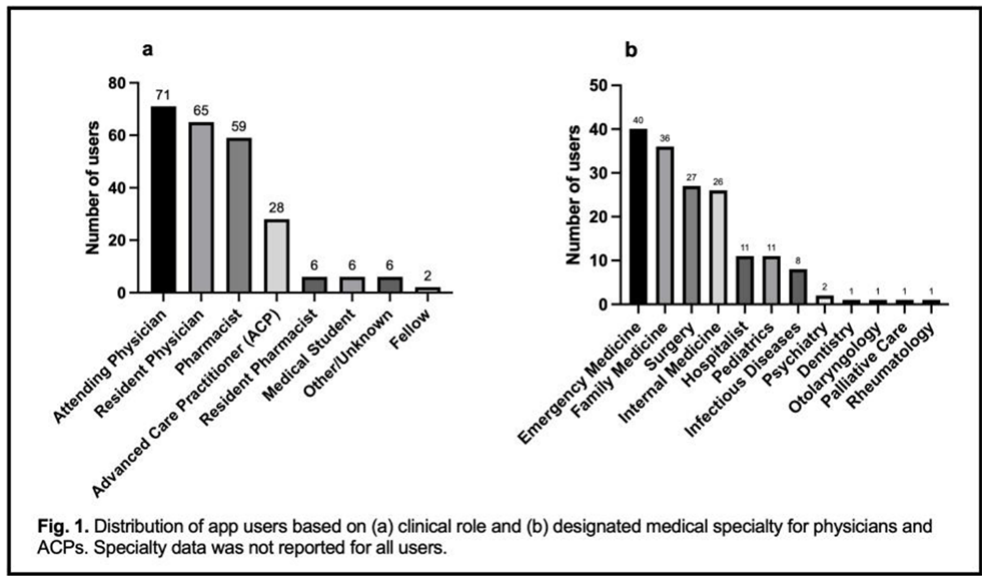
AMS App Users by Clinical Role and Provider Specialty

**Figure:**
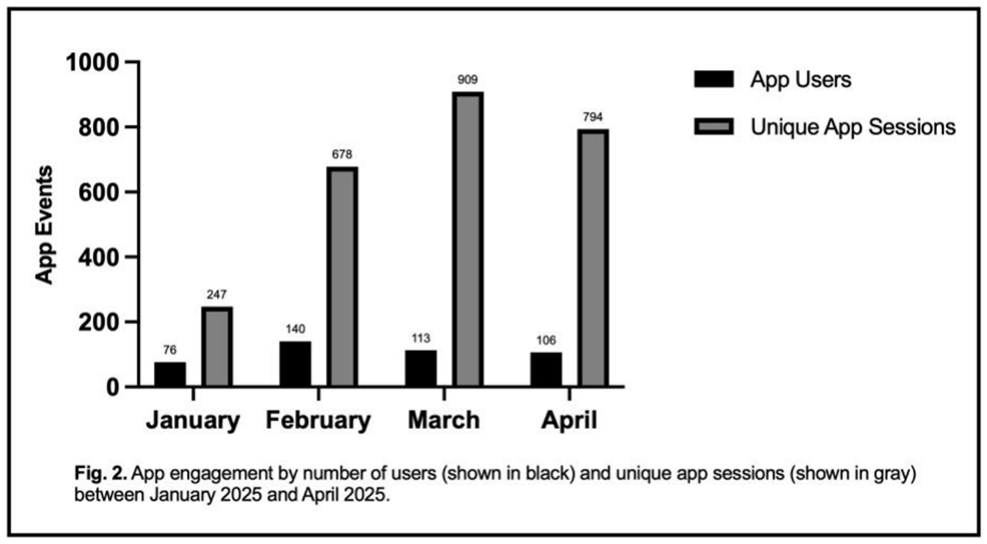
AMS App Users and Unique Sessions by Month

**Methods:**

A multi-disciplinary team, including staff from infectious diseases, pharmacy, and infection prevention, developed an AMS app using the Eolas Medical platform. The app housed a range of organization-specific resources including antibiotic treatment guidelines, provider education, microbiology guides, and other tools. Most resources on the app were previously available to clinicians but were difficult to locate online or were behind a firewall. The app was launched and promoted by physician and pharmacy leadership in January 2025 and made available on mobile phones and through a weblink. We measured uptake and usage of the app following the launch and evaluated access to AMS resources pre- and post-launch using Student’s t-tests.

**Figure:**
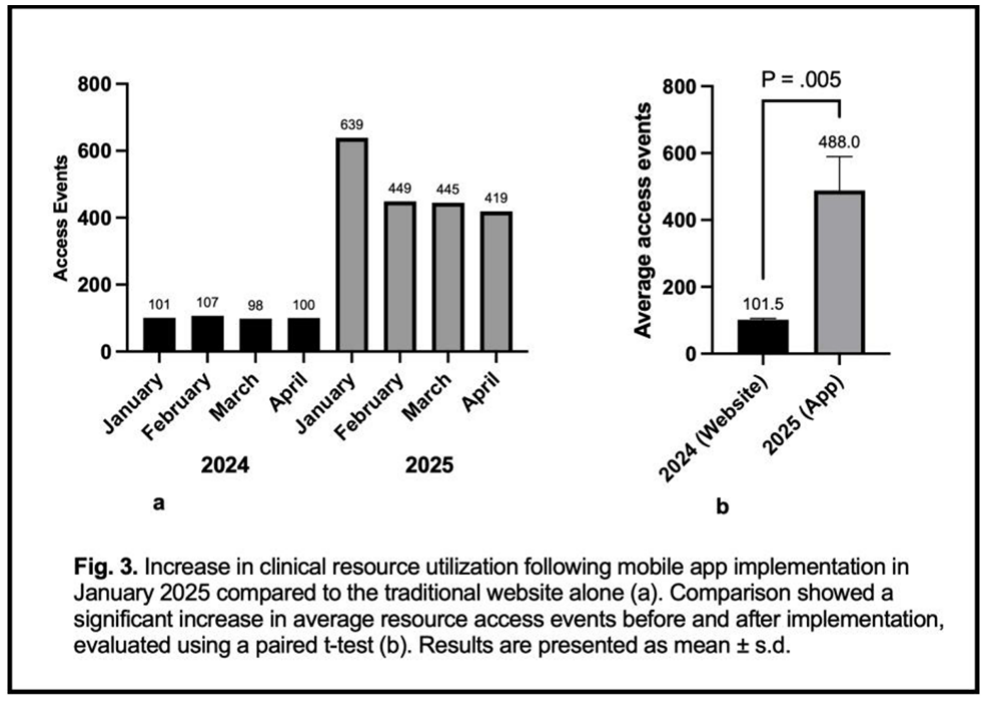
Access to AMS Resources by Month Pre- and Post-Launch of App

**Figure:**
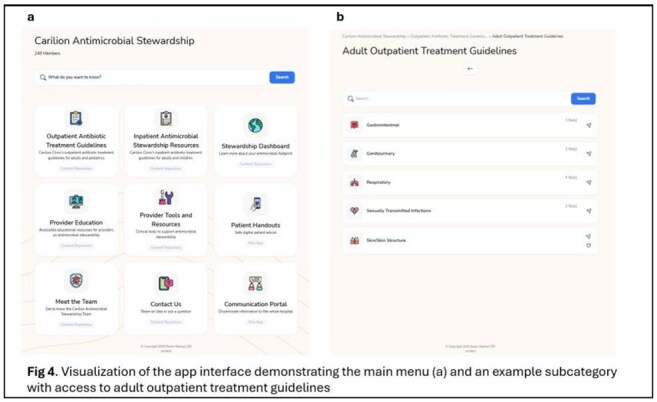
AMS App Interface

**Results:**

In the first four months of implementation, 244 users affiliated with our healthcare system created an Eolas account and accessed the AMS app. 68% of these were providers (physicians or advanced care practitioners) and 27% were pharmacists. Among providers, the specialties with the greatest number of users were emergency medicine (24%), family medicine (22%), surgery (16%), and internal medicine (16%) (Fig. 1). The average number of unique app users per month was 109 and the average number of sessions was 657 (Fig. 2).

Since the launch, AMS resources were accessed an average of 488 times a month compared to 102 times per month in the same four-month period in 2024 when the app was not available (p = 0.005) (Fig. 3).

**Conclusion:**

The app increased usage of AMS resources by clinicians in key specialties, suggesting apps may bolster AMS programs by making resources more accessible. Continued support may be needed to systematize clinician engagement with the platform. We plan to enhance the app by integrating clinical decision support tools, including AI, into the platform to maximize functionality.

**Disclosures:**

All Authors: No reported disclosures

